# The effect of preoperative clindamycin in reducing early oral implant failure: a randomised placebo-controlled clinical trial

**DOI:** 10.1007/s00784-022-04701-9

**Published:** 2022-09-13

**Authors:** Gorka Santamaría Arrieta, Fabio Rodríguez Sánchez, Carlos Rodriguez-Andrés, Luis Barbier, Iciar Arteagoitia

**Affiliations:** 1 Faculty of Medicine and Nursing, University of the Basque Country, Leioa, Spain; 2 Biocruces Bizkaia Health Research Institute, Barakaldo, Spain; 3 Department of Oral Health Sciences, Section Periodontology, Catholic University of Leuven and University Hospitals Leuven, Louvain, Belgium

**Keywords:** Clinical trial, Clindamycin, Implant failures, Postoperative infections, Antibiotic prophylaxis

## Abstract

**Objectives:**

To assess the effect of preoperative oral clindamycin in reducing early implant failure in healthy adults undergoing conventional implant placement.

**Materials and methods:**

We conducted a prospective, randomised, double-blind, placebo-controlled clinical trial in accordance with the ethical principles and Consolidated Standards of Reporting Trials statement. We included healthy adults who underwent a single oral implant without previous infection of the surgical bed or the need for bone grafting. They were randomly treated with a single dose of oral clindamycin (600 mg) 1 h before surgery or a placebo. All surgical procedures were performed by one surgeon. A single trained observer evaluated all patients on postoperative days 1, 7, 14, 28, and 56. Early dental implant failure was defined as the loss or removal of an implant for any reason. We recorded the clinical, radiological, and surgical variables, adverse events, and postoperative complications. The study outcomes were statistically analysed to evaluate differences between the groups. Furthermore, we calculated the number required to treat or harm (NNT/NNH).

**Results:**

Both the control group and clindamycin group had 31 patients each. Two implant failures occurred in the clindamycin group (NNH = 15, *p* = 0.246). Three patients had postoperative infections, namely two placebo-treated and one clindamycin-treated, which failed (relative risk: 0.5, CI: 0.05–5.23, absolute risk reduction = 0.03, confidence interval: − 0.07–0.13, NNT = 31, CI: 7.2–∞, and *p* = 0.5). One clindamycin-treated patient experienced gastrointestinal disturbances and diarrhoea.

**Conclusions:**

Preoperative clindamycin administration during oral implant surgery in healthy adults may not reduce implant failure or post-surgical-complications.

**Clinical relevance:**

Oral clindamycin is not efficacy.

**Trial registration:**

The present trial was registered (EudraCT number: 2017-002,168-42). It was approved by the Committee for the Ethics of Research with Medicines of Euskadi (CEIm-E) on 31 October 2018 (internal code number: 201862) and the Spanish Agency of Medicines and Medical Devices (AEMPS) on 18 December 2018.

## Introduction

Oral implants are considered a predictable and safe treatment modality for the rehabilitation of missing teeth [1]. However, there are reports on oral implant failure [2]). Postoperative infections following the bacterial contamination of the implant bed during surgery are a significant cause of early implant failure, as well as being associated with late implant failure [3].The periodontal status of a patient before implant placement is also associated with early implant loss. In 2012, Pjetursson [4] reported that residual pockets (probing pocket depth, PPD ≥ 5 mm) at the end of active periodontal therapy represent a significant risk of developing peri-implantitis and implant loss in periodontitis susceptible patients. In addition, instrumentation may be associated with material wear, and this phenomenon should be taken into consideration to control peri-implant inflammatory reactions that may lead to implant failures [5].

Consequently, researchers have advocated prophylactic antibiotics to prevent postoperative infections and oral implant failures [6]. However, the irrational use of antibiotic therapy could potentially cause adverse reactions, thus warranting consideration. Adverse events related to the use of antibiotics range from diarrhoea to life-threatening allergic reactions, in addition to the development of bacterial resistance [7, 8]

Prophylactic antibiotics are generally recommended during surgery for patients at risk of infectious endocarditis, with reduced host response, surgery at infected sites, extensive and prolonged surgeries, and the implantation of large foreign materials [9]. However, the use of prophylactic antibiotics in oral implant surgery remains controversial. Some studies have demonstrated reduced early implant failure following preoperative antibiotic administration in appropriate doses [7, 10–12]. By contrast, several studies do not recommend their use [13–18]

Recent systematic reviews and meta-analyses of randomised clinical trials (RCT) [19–22] reported that a single antibiotic prophylaxis dose reduces the incidence of early implant failure. The Consensus Report by the Italian Academy of Osseointegration on the use of antibiotics and antiseptic agents in implant surgery advocates the administration of a unique dose of antibiotics in straightforward implant cases, combined with chlorhexidine [23].

Despite current studies published in this field, there is a lack of consensus among clinicians while prescribing antibiotics in oral implant surgery. Moreover, their prescriptions may not frequently follow evidence-based recommendations [24, 25].

Recent surveys have confirmed that more than half of the dentists who routinely place implants prescribe antibiotics [26–28]. In Italy, approximately 84% of the patients are routinely prescribed prophylactic antibiotics in conjunction with oral implant surgery [29].

Amoxicillin is one of the most extensively studied and prescribed antibiotics [17–20]. A recent meta-analysis focused on the effect of antibiotic regimens in early implant failure, including 11 RCTs, all of which used amoxicillin as the antibiotic of choice or clindamycin in case of an allergy [20].

Clindamycin has appeared as one of the choices for oral implant surgery according to multiple surveys performed in the last few years among oral care professionals [26, 30–36]. In a survey performed in Spain, 58% of the respondents selected clindamycin as the alternative in cases of an allergy to amoxicillin [36].

A single 600-mg dose of clindamycin 1 h preoperatively was reported as one of the routinely prescribed regimens for implant surgery in Sweden [37]. Moreover, it was an alternative antibiotic regimen most prescribed for penicillin allergies in the UK (18% of the participants) [27]. Recent practical antibiotic prescription guidelines in Belgium [38, 39] and Italy [23] also recommend a single dose of 600-mg clindamycin prior to surgery.

Clindamycin has been studied to this purpose previously [12, 40]. Previous studies have compared between single preoperative doses of clindamycin or with penicillin in patients undergoing bone graft procedures [41, 42].

According to several researchers and national guidelines, clindamycin is principally prescribed for patients allergic to amoxicillin to prevent the loss of dental implants. However, no clinical trial has been published yet to support its efficacy neither in allergic nor in non-allergic patients to penicillin. Therefore, we intended to conduct an RCT that provides information regarding this. As a consequence, we aimed to assess the effect of a single 600-mg dose of oral clindamycin administered 1 h pre-operatively in reducing early implant failure and on post-surgical complications in healthy adults undergoing a conventional implant installation.

The null hypothesis was postulated as follows: there are no differences in the cumulative incidence of implant failures following an oral implant surgery in medically and periodontally healthy adults and straightforward conditions upon administering a single 600-mg dose of oral clindamycin 1 h preoperatively versus placebo.

## Materials and methods

### Trial design and ethical aspects

This study was a prospective, randomised, parallel, double-blind, and placebo-controlled clinical trial (EudraCT number: 2017–002,168-42). It was approved by the Committee for the Ethics of Research with Medicines of Euskadi (CEIm-E) on 31 October 2018 (internal code number: 201862) and the Spanish Agency of Medicines and Medical Devices (AEMPS) on 18 December 2018. This study was conducted in accordance with ethical principles, including the World Medical Association Declaration of Helsinki (World Medical Association, 2013) and the Consolidated Standards of Reporting Trials (CONSORT) statement [43].

### Participants

The trial was conducted at the Dental Clinic of the Postgraduate in Oral Implantology and Microsurgery at the University of the Basque Country (Leioa, Spain). Patients were only eligible for the study when they were healthy adults (at least 18 years old) who had attended the dental clinic and were indicated for the placement of a single oral implant, without a previous surgical site infection or bone-graft procedure. We performed consecutive sampling of the accessible population.

Participants were excluded before randomisation for the following reasons: allergy to any drugs used in the trial, decompensated systemic pathologies (cardiac, respiratory, endocrine, metabolic, hepatic, haematological, the risk of bacterial endocarditis, or immunosuppression), valvular or orthopaedic prostheses, and a history or use of bisphosphonates, anticoagulants, or antiplatelet agents. Moreover, we excluded participants whose amannesis reports that they have had been irradiated in the cervical and maxillofacial territory, were pregnant, suspected of being pregnant or breastfeeding, or with a history of antibiotic-associated ulcerous colitis. All participants underwent a radiographical assessment at the implant site, as stated in the clinic’s protocol. Those requiring bone graft treatment were excluded from the trial. We recorded the PPD and bleeding on probing to determine their periodontal status. Only healthy participants were included in this study.

Participants were excluded following randomisation upon their request, by an abandonment of the trial, by loss to follow-up, and/or antibiotic consumption in the last 15 days prior to the surgery.

### Interventions

All participants in the test group were administered a single dose of 600-mg clindamycin (two capsules of 300 mg) 1 h before surgery. Those in the control group received two capsules of placebo 1 h before surgery. The placebo and antibiotics had similar characteristics. The participants were provided an envelope with the medication or placebo at the dental clinic 1 h before the surgery.

All surgical procedures were performed by one surgeon with an extensive experience in oral implant surgery. The surgeries commenced with the following anaesthetic technique: anaesthetic block of the area using articaine (4%) with epinephrine 1:1000.000, using a mandibular block technique for implant placement in the mandible, and an infiltrative technique in the maxilla. A full-thickness mucoperiosteal flap was made via a crestal incision in the edentulous section and an intrasulcular incision in the adjacent teeth. Releasing incisions were made only on the highly resorbed ridges or in the presence of marked bone concavities. Straumann Roxolid® (TI-Zr) SLActive® (Sand-blasted large grift acid-etched) (Basel, 4002 Switzerland) implants were placed. The surgeon inserted a 1.8 mm Straumann Tissue Level Standard Plus® (TL) polished neck implant in non-aesthetic zone. Straumann Bone Level Tapered ® (BLT) type implants were inserted in the aesthetic areas of the maxilla. The manufacturer-recommended drilling sequence was used for both types of implants. None of the participants underwent a second-stage implant surgery. The bone width and available bone height determined the diameter (3.3 or 4.1 mm) and length of the implant (8, 10, or 12 mm), and therefore the drilling sequence. Al implants were placed freehand. Following the implant placement, the implant insertion torque was measured using a Straumann® ratchet wrench with a dynamometer (Nos. 046.119 and 046.049). In all cases, primary closure of the flap was done using a 5–0 non-resorbable. Polyester monofilament suture with a single interrupted suturing technique.

Postoperative pain management consisted of 600-mg ibuprofen or 1-g paracetamol prescribed every 8 h on the days the participants considered it necessary, but no more than 4 days.

The clindamycin and placebo capsules were presented in blister packs, individually packed, and labelled to maintain blinding. The samples were labelled with the sample number, protocol code, the number of units, dosage form, the route of administration, and expiration date.

The planned antibiotic was one tablet (875/125 mg) of amoxicillin/clavulanic acid administered every 8 h for 7 days. One of the inclusion criteria was no allergy to amoxicillin or clavulanic acid.

### Outcomes

A single experimented observer evaluated all patients on post-operative days 1, 7, 14, 28, and 56. The primary outcome was implant failure. Failure was defined as the loss or removal of an implant for any reason.

The secondary outcome was any clinical or radiographic signs indicative of an infection. Were recorded, peri-implant radiolucency, manual mobility, and Osstell® resonance frequency analysis (Implant stability quotient, ISQ < 60, including suppuration, fistula, abscess, osteomyelitis, and fever > 38 °C. Furthermore, we assessed postoperative pain, localised inflammation, bleeding, and intraoral and extraoral erythema using the visual analogue scale.

In addition, we recorded the adverse reactions.

### Sample size

We calculated the sample size using the statistical program WinEpi: Working in Epidemiology; http://www.winepi.net/f108.php > 18% differences in infection or implant failure between the control and treatment groups were considered clinically relevant. Nolan et al. (2014) reported on an 18% cumulative incidence of failure in patients treated with a placebo. Two groups with 31 patients each were required for a 95% confidence level (CI), a power of 80%, the probability of implant failure of 18%, and 0% with placebo and antibiotics, respectively. Consequently, a treatment group (TG) and control group (CG) with 31 other implants each were considered necessary. The sample consisted of 62 participants who freely provided their informed consent.

### Randomisation

From the total sample, we performed a block-switching restricted randomisation comprising a block length of four patients, with a similar probability (0.5) of allocation to each treatment within the block (two patients for each treatment within each block). Randomisation was performed using the statistical program STATA® 15 (College Station, TX, 77,845, USA).

### Allocation concealment

The participants were assigned after determining their eligibility as per the inclusion criteria and obtaining their informed and written consent to participate. An assistant outside the study prepared sealed and numbered envelopes with the antibiotic or placebo to be administered, according to the instructions. Each number corresponded to the treatment assigned during randomisation, and each patient was successively assigned the corresponding treatment number. One of the researchers delivered the treatment in a closed envelope 1 h before the implant placement. All the professionals directly in contact with the participants and the participants themselves were unaware of the treatment.

### Blinding

We performed randomisation and allocation concealment with double blinding as follows: neither the participants nor the expert who placed the implant were aware of the treatment received. The professional who evaluated for infection or the loss of implant was also unaware of the treatment.

### Statistical analysis

STATA ® 15 software (College Station, TX, 77,845, USA) was used for the intention-to-treat data analysis. We calculated the variances for each variable, and assessed the association between treatment groups and different variables using the Student’s *t* test and Fisher’s exact test for continuous and categorical variables, respectively. The treatment effect and accuracy were estimated using 95% CI. Furthermore, we calculated the absolute risk reduction or increase (ARR or ARI), relative risk (RR), the number needed to treat or harm (NNT or NNH) for implant failure, postoperative infection, adverse events, and complications (infections or implant failure). We analysed the hypotheses of equal risk for implant failure or postoperative infections between the treatment and control groups.

This study was conducted in compliance with the CONSORT checklist.

## Results

### Participant flow

**Fig. 1:**
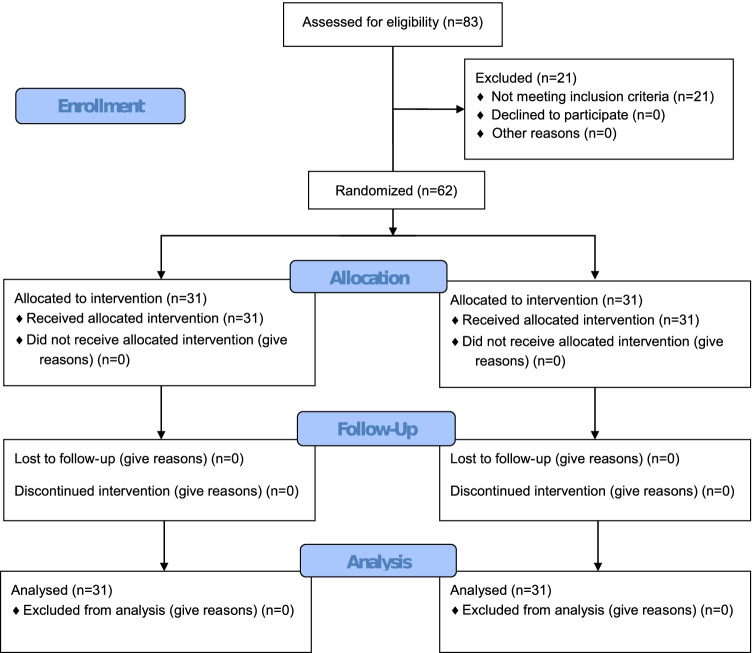
Flow chart of the enrolment process

Figure 1 is a flow diagram depicting the number of participants who were randomly assigned, received the intended treatment, and were analysed for each group as well as the losses and exclusions following randomisation.

### Recruitment

Participant recruitment began in October 2019 and ended in June 2021, and each participant was followed-up until day 56 post-surgery. The trial was temporarily halted owing to the pandemic caused by coronavirus disease 2019 and ended upon monitoring all included participants on day 56 post-surgery (August 2021).

### Baseline data

Table 1 summarises the baseline demographic and clinical characteristics of each group.

**Table 1:** Participant characteristics

Variable	Clindamycin group	Control group	Overall
	(*n* = 31)	(*n* = 31)	(*n* = 62)
Gender			
Male	14 (45.2%)	8 (25.8%)	22 (35.5%)
Female	17 (54.8%)	23 (74.2%)	40 (64.5%)
Age	49.7 (9.4)	47.5 (10.7)	48.6 (10.1)
Smokers			
Yes	7 (22.6%)	6 (19.4%)	13 (20.9%)
No	24 (77.4%)	25 (80.6%)	49 (79.1%)
Cigarettes per day			
0	24 (77.4%)	25 (80.6%)	49 (79.1%)
< 10	3 (9.6%)	4 (12.9%)	7 (11.3%)
10–20	2 (6.5%)	2 (6.5%)	4 (6.4%)
> 20	2 (6.5%)	0 (0%)	2 (3.2%)
Contraceptives			
Yes	1 (3.3%)	4 (12.9%)	5 (8.1%)
No	30 (99.7%)	27 (87.1%)	57 (91.9%)
Implant location			
Upper premolar	4 (12.9%)	6 (19.3%)	10 (16.1%)
Upper molar	6 (19.3%)	6 (19.3%)	12 (19.4%)
Lower premolar	0	2 (6.5%)	2 (3.2%)
Lower molar	21 (67.8%)	17 (54.9%)	38 (61.3%)
Implant type			
Bone level	11 (35.5%)	12 (38.7%)	23 (37.1%)
Tissue level	20 (64.5%)	19 (61.3%)	39 (62.9%)
8 mm	2 (6.4%)	4 (12.9%)	6 (9.6%)
10 mm	29 (93.6%)	27 (87.1%)	56 (90.4%)
Surgery duration	14.5 (5.1)	15.3 (6.1)	14.9 (5.6)

### Analysed numbers

Thirty-one participants each from the clindamycin-treated and control groups were eventually included in the analysis. The analysis was performed in all cases using the original assigned groups.

### Outcomes and estimation

Overall, two implant failures occurred in participants treated with clindamycin (RR: not estimable, ARI = 0.06; CI: − 0.03–0.16, NNH = 15.5; and CI: 6–∞). The ARI indicated that 6% of the patients would experience implant failure under clindamycin treatment and would not have received placebo. The NNH predicted that for every 15 participants treated with clindamycin, one implant failure would occur beyond those that would have happened under placebo treatment. Nevertheless, there were no significant differences between the groups (*p* = 0.246).

Three patients suffered postoperative infections, and two of them were administered rescue antibiotic treatment. By contrast, the remaining patient did not receive the rescue antibiotic owing to implant failure and removal. Both participants who received the rescue antibiotic belonged to the control group, whereas the participant with implant failure belonged to the clindamycin group (RR: 0.5; CI: 0.05–5.23, ARR = 0.03; CI: − 0.07–0.13, NNT = 31; and CI: 7.2–∞). The ARR suggested that 3.2% of the patients would not experience postoperative infections under clindamycin treatment, which they would experience under placebo. The NNT indicated that 31 patients would need to be treated with clindamycin to prevent one patient from suffering a postoperative infection. However, there were no significant differences between the groups (*p* = 0.554).

Considering the occurrence of postoperative infections or oral implant failures per participant as the overall complications, there were two participants in each treatment group with complications (RR = 1; CI: 0.15–6.66, ARR = 0; CI: − 0.12–0.12, NNT: not estimable, and *p* = 0.999).

### Ancillary analyses

There were no significant relationships between the recorded variables and implant failure or adverse reactions (Table 2). We did not identify significant associations between the variables and postoperative infection, except for the implant type (*p* = 0.047) and location (*p* = 0.048). The implant location was borderline non-significant for postoperative infection (*p* = 0.055). Furthermore, there were no significant differences (*p* > 0.05) between the treatment groups and the following variables: suppuration, fistula, abscess, osteomyelitis, fever > 38 °C, postoperative pain, bleeding, localised inflammation, extraoral erythema, intraoral erythema, peri-implant radiolucency on day 56, and ISQ value < 60 on day 56 (Table 3).

**Table 2:** Major outcome variables

Variable	Implant Failure			Postoperative infections			Adverse reactions		
	Failures proportion	RR (95% CI)	*p*-value	Infection proportion	RR (95% CI)	*p*-value	Adverse reactions proportion	RR (95% CI)	*p*-value
Treatment group									
**Clindamycin**	**2/31**	**NS**	**0.246**	**1/31**	**0.5 (0.05–5.23)**	**0.5**	**1/31**	**NS**	**0.5**
Placebo	0/31			2/31			0/31		
Genre									
Male	1/22	1	0.588	0/22	NS	0.261	1/22	NS	0.355
Female	1/40			3/40			0/40		
Implant type									
Bone level	2/23	NS	0.134	3/23	NS	0.047	0/23	NS	0.629
Tissue level	0/39			0/39			1/39		
Implant length									
8 mm	0/6	NS	0.814	1/6	4.6 (0.5–44.1)	0.267	0/6	NS	0.903
10 mm	2/56			2/56			1/56		
Implant location									
Maxilla	2/22	NS	0.048	3/22	NS	0.055	0/22	NS	0.999
Mandible	0/40			0/40			1/40		
Smoker									
Yes	1/13	1	0.378	0/13	NS	0.487	0/13	NS	0.790
No	1/49			4/49			1/49		
Contraceptives									
Yes	0/5	NS	0.844	0/5	NS	0.774	0/5	NS	0.919
No	2/57			3/57			1/57		

^*^p values were obtained by the Fisher’s exact test

^†^*CI* confidence interval: *NS* not estimable

**Table 3:** Outcome variables

Follow-up	Treatment group	Suppuration	Fistula	Abscess	Osteomyelitis	Fever > 38 °C	Post-operative pain	Localised inflammation	Bleeding	Extraoral erythema	Intraoral erythema	Peri-implant radiolucency	ISQ value < 60
Day 1	Clindamycin group	0/31	0/31	0/31	0/31	0/31	0.451	1.419	0	0.096	0.677	-	-
	Control group	0/31	0/31	0/31	0/31	0/31	0.677	1.483	0.096	0.032	0.774	-	-
	*p* value	-	-	-	-	-	0.2315	0.7992	0.179	0.531	0.6723	-	-
Day 7	Clindamycin group	0/31	0/31	0/31	0/31	0/31	0.064	0.483	0	0.032	0.032	-	-
	Control group	0/31	0/31	0/31	0/31	0/31	0.064	0.612	0	0	0.032	-	-
	*p* value	-	-	-	-	-	0.9999	0.4971	-	-	0.9999	-	-
Day 14	Clindamycin group	0/31	0/31	0/31	0/31	0/31	0	0.032	0	0.064	0	-	-
	Control group	0/31	0/31	0/31	0/31	0/31	0.064	0.064	0.032	0	0	-	-
	*p* value	-	-	-	-	-	0.3213	0.5615	0.3213	0.1607	-	-	-
Day 28	Clindamycin group	0/31	1/31	1/31	0/31	0/31	0	0.032	0.258	0	0.096	-	-
	Control group	1/31	1/31	0/31	0/31	0/31	0.064	0.032	0	0	0.064	-	-
	*p* value	0.5	0.754	0.5	-	-	0.3213	0.9999	0.2063	-	0.7826	-	-
Day 56	Clindamycin group	0/31	0/31	0/31	0/31	0/31	0	0.032	0	0	0	1/31	2/31
	Control group	0/31	0/31	0/31	0/31	0/31	0.064	0.032	0	0	0	1/31	0/31
	*p* value	-	-	-	-	-	0.3213	0.9999	-	-	-	0.9999	0.151

The rate is provided for continuous variables, whereas *p* values were obtained by the Student’s *t* test

The mean is provided for categorical variables, whereas *p* values were obtained using the Fisher’s exact test

### Harms

Only one clindamycin-treated participant experienced adverse events (gastrointestinal disorders and diarrhoea), thus yielding no significant differences between the groups (RR: not estimable, ARI = 0.03; CI: − 0.05–0.11, NNH = 31; CI: 8.5–∞, and *p* = 0.5).

## Discussion

### Interpretation

The present clinical trial demonstrated that a single 600-mg preoperative dose of oral clindamycin did not differ from placebo in preventing oral implant failures or postoperative infections following oral implant surgery under ordinary conditions in healthy adults.

Several reviews and meta-analyses have demonstrated that a single 2-g preoperative dose of oral amoxicillin may be effective against oral implant surgery; nonetheless, the effectiveness of this treatment was questionable (NNT varied from 25 to 77), and the use of prophylactic antibiotics in oral implant surgery remains controversial [2, 9, 19, 20, 44, 45].

Recent publications have described a wide variation in antibiotic regimens and types prescribed in different countries [24] for oral implant surgery, but with a clear preference for amoxicillin and other drugs from the penicillin group. Variance in antibiotic consumption between countries has been associated with higher levels of bacterial resistance to penicillin, amoxicillin, metronidazole, clindamycin, and tetracycline [46]. Antimicrobial resistance poses a real threat to the health and well-being of humans and animals globally (Report to the Secretary-General of the United Nations) [47]. Therefore, it is imperative to investigate the necessity of using these antibiotics routinely.

Clindamycin is frequently prescribed for penicillin-allergic patients as an alternative to oral surgery; however, researchers have recently reported on the lack of high-quality clinical evidence for its use in oral implant surgery [28, 36, 48–50]. Clindamycin is a broad-spectrum antibiotic with activity against aerobic, anaerobic, and β-lactamase-producing pathogens. It has been used for several years as a prophylactic treatment during dental procedures to prevent endocarditis [51]. However, the American Heart Association stopped recommending clindamycin as an alternative antibiotic for prophylaxis against infective endocarditis since last year [52]. The American Dental Association [53] no longer recommends clindamycin for patients with a history of penicillin allergy owing to the frequent and serious adverse effects associated with clindamycin, compared with other prophylactic options, including *C. difficile* infections.

The present clinical trial represented a further step towards understanding the lack of evidence supporting clindamycin’s prophylactic use.

Moreover, the trial can likely investigate the controversy regarding the association between oral implant failures, penicillin allergy, and the use of clindamycin (Salgado-Peralvo et al., 2021a; Salgado-Peralvo et al., 2021b). Considering the exclusion of all participants allergic to penicillin from the trial, we could delimitate the correlation between the possible higher occurrence of implant failures and clindamycin use. Hypothetically, the role of unknown genetic factors in penicillin-allergic participants was excluded from this equation.

The low incidence of postoperative infections may be the supposed outcome of standard antiseptic-sterile surgical measures, in combination with the performance of an expert surgeon and limited surgical duration. Nevertheless, the postoperative infection rates (3.2% for the clindamycin group and 6.4% for the control group) were considerably higher but similar to those reported in other clinical trials performed with alternate antibiotics (2.2% for the amoxicillin group and 3.1% for the control group) (Rodríguez Sánchez et al., 2018).

### Limitations

It is crucial to consider the underpowering of the study while interpreting the clinical outcomes.

Unfortunately, the participants did not undergo any allergy test for penicillin; therefore, they could have been allergic to penicillin.

The intention-to-treat analysis and the use of rescue antibiotics could have masked the development of a serious infection and the eventual failure of the implant.

### Generalizability

Our findings were applicable to the population from which the individuals were included in the trial and can be generalised to similar populations There were no limitations in the external validity and applicability of this study to such populations; in this specific condition, oral implant surgery was performed by an expert surgeon and accomplished in healthy adults with completely healed implant sites, without preoperative infections or the need for bone augmentation procedures.

In conclusion, the use of preoperative clindamycin in oral implant surgery under straightforward conditions in healthy adults may not be beneficial in reducing oral implant failures. Further clinical research is needed to assess if clindamycin is beneficial in complex surgeries. In all cases, the risk–benefit ratio should be cautiously considered to maintain balance in our clinical decisions.

## Data Availability

The data that support the findings of this study are available from the corresponding author upon reasonable request.
